# Prediction of Multi-Target Networks of Neuroprotective Compounds with Entropy Indices and Synthesis, Assay, and Theoretical Study of New Asymmetric 1,2-Rasagiline Carbamates

**DOI:** 10.3390/ijms150917035

**Published:** 2014-09-24

**Authors:** Francisco J. Romero Durán, Nerea Alonso, Olga Caamaño, Xerardo García-Mera, Matilde Yañez, Francisco J. Prado-Prado, Humberto González-Díaz

**Affiliations:** 1 Department of Organic Chemistry, Faculty of Pharmacy, University of Santiago de Compostela (USC), Santiago de Compostela 15782, Spain; E-Mails: fcojavier.romero@rai.usc.es (F.J.R.D.); nerea.alonso@rai.usc.es (N.A.); molga.caamano@usc.es (O.C.); fprado@uqroo.mx (F.J.P.-P.); 2 Department of Pharmacology, University of Santiago de Compostela (USC), Santiago de Compostela 15782, Spain; E-Mail: matilde.yanez@usc.es; 3 Biomedical Sciences Department, Health Sciences Division, University of Quintana Roo (UQROO), Chetumal 77039, Mexico; 4 Department of Organic Chemistry II, Faculty of Science and Technology, University of the Basque Country UPV/EHU, Leioa 48940, Spain; 5 IKERBASQUE, Basque Foundation for Science, Bilbao 48011, Spain

**Keywords:** CHEMBL, neuroprotective agents, rasagiline derivatives, asymmetric synthesis, multi-target drugs, molecular information measures, Shannon entropy, Markov chains, moving averages

## Abstract

In a multi-target complex network, the links (*L*_ij_) represent the interactions between the drug (*d*_i_) and the target (*t*_j_), characterized by different experimental measures (*K*_i_, *K*_m_, IC_50_, *etc*.) obtained in pharmacological assays under diverse boundary conditions (*c*_j_). In this work, we handle Shannon entropy measures for developing a model encompassing a multi-target network of neuroprotective/neurotoxic compounds reported in the CHEMBL database. The model predicts correctly >8300 experimental outcomes with Accuracy, Specificity, and Sensitivity above 80%–90% on training and external validation series. Indeed, the model can calculate different outcomes for >30 experimental measures in >400 different experimental protocolsin relation with >150 molecular and cellular targets on 11 different organisms (including human). Hereafter, we reported by the first time the synthesis, characterization, and experimental assays of a new series of chiral 1,2-rasagiline carbamate derivatives not reported in previous works. The experimental tests included: (1) assay in absence of neurotoxic agents; (2) in the presence of glutamate; and (3) in the presence of H_2_O_2_. Lastly, we used the new Assessing Links with Moving Averages (ALMA)-entropy model to predict possible outcomes for the new compounds in a high number of pharmacological tests not carried out experimentally.

## 1. Introduction

Entropy measures are universal parameters useful to codify biologically relevant information in many systems. In the 1970’s Bonchev and Trinajstic *et al*. published works about the use of Shannon’s entropy to calculate a structural information parameter [1,2,3,4]. Kier published another seminar works on the use of Shannon’s entropy to encoding molecular structure in Cheminformatics studies in 1980 [4]. Many other authors used Shannon’s entropy parameters for the same purpose on small molecule structure [5,6,7,8,9,10]. Graham *et al*. [11,12,13,14,15,16] used entropy measures to study the information properties of organic molecules. Entropy information measures were used to describe proteins [17,18], DNA sequences [19], protein networks [20], and magnetic resonance outcomes [21]. The software MARCH-INSIDE (MI) uses the theory of Markov chains to calculate the parameters θ_k_(G). These values are the Shannon entropies of order k^th^ of a graph G. The θ_k_(G) values are useful quantify information about the structure of molecular systems [22]. The graph G represents a complex molecular system as a network of nodes interconnected by links (*L*_ij_ = 1) or not connected (*L*_ij_ = 0). MI algorithm associates a Markov matrix to the graph G in order to define the probabilities of interactions (ties or relationships) between nodes. These entropy parameters θ_k_(G) can be calculated for many types of systems (molecular or otherwise). We have studied small molecules, RNA secondary structures, protein sequences, viral surfaces, cerebral cortex networks, metabolic networks, host-parasite networks, world trading networks, social networks, *etc*. In molecules, we know the information about links *L*_ij_ (covalent bonds, hydrogen bonds, spatial contacts, *etc*.) beyond any reasonable doubt. However, we can use the information (θ_k_(G) values)of the system to predict interactions with other systems in a network of a higher-structural level. For instance, we use the θ_k_(G) values of drugs and targets structure to predict drug–target interactions (links) in drug–target network. In other cases, linking patterns change, are not known, or we find contradictory information. This is the case of the existence of different relationships between nodes in biological webs or social networks. In these cases, we can use the θ_k_(G) values of known networks to find models useful to predict links in new networks [23,24,25].

On the other hand, the discovery of new drugs for the treatment of neurodegenerative diseases such as Alzheimer’s, Parkison’s, and Huntington’s disease, Friedreich ataxia and others, is an important goal of medicinal chemistry [26,27,28,29]. The genes causing hereditary forms of some of these diseases have been identified but the molecular mechanisms of the neuronal degeneration have not been totally understood yet [30]. This picture, and some disappointing results in clinical trials, makes interesting the prediction of drug candidates with computational techniques [31,32]. In order to design these computational models we need to process chemical information from public databases. These databases have accumulated immense datasets of experimental results of pharmacological trials for many compounds. For instance, CHEMBL [33,34] is one of the biggest with more than 11,420,000 activity data for >1,295,500 compounds, and 9844 targets. This huge amount of information offers a fertile field for the application of computational techniques [34,35].

The analysis of all this data is very complex due to the presence of multi-target, multi-output, and multi-scale information. Multi-target complication emerges due to the existence of compounds with multiple targets [36,37,38]. This led to the formation of complex networks of drug–target interactions. We can represent drug–target networks as a graph with two types of nodes drugs (*d*_i_) and targets (*t*_j_) interconnected by links (*L*_ij_). Barabasi *et al*. [39], constructed a drug–target network based on Food and Drug Administration (FDA) drugs and proteins linked by drug–target binary associations. Csermely *et al*. [40], reviewed the use of networks, including drug–target networks, for drug discovery.

Multi-output feature refers to the necessity of prediction of different experimental parameters (IC_50_, *K*_i_, *K*_m_, *etc*.) to decided whether two nodes (drug and target) interact (*L*_ij_ = 1) or not (*L*_ij_ = 0). Multi-scaling refers to the different structural levels of the organization of matter. In this case, the input variables quantify molecular information (drugs structure) and macromolecular information (targets). They have to quantify also cellular (cellular targets) and organism information (specie that express the target). In these models we have a high number of assays carried out in very different conditions (*c*_q_) like time, concentrations, temperature, cellular targets, tissues, organisms, *etc*. In a recent work, we combined the θ_k_(G) values calculated with MI and the idea of Moving Average (MA) operators with a similar purpose [41]. In time series analysis the MA operators are average values of characteristic of the system for different seasons. In fact, MA models became popular after the initial works of Box and Jenkins [42]. In time series analysis, MA models may combine other operators I = Integrated, AR = Autoregressive, N = Non-linear operators, or X = Exogenous effects. In this sense, others models have emerged combining different operators: ARMA, ARIMA, VARIMA, ARIMAX, NARMA, *etc*. In multi-output modeling, we calculate the MA operators as the average of the property of the system (molecular descriptors or others) for all drugs or targets with a specific response in one assay carry out under a sub-set of conditions (*c*_j_). Consequently, our MA operator is not acting over a time domain but over a sub-set of conditions of the pharmacological assays. The idea of application of MA operators to other domains different from time is gaining adepts due to its advantages. For instance, Botella-Rocamora *et al*. [43] developed a model for disease mapping using spatial Box–Jenkins operators with the form of MAs, to define dependence of the risk of a disease to occur. In our models, we use MA in relation with properties of nodes of networks (drugs, proteins, reactions, laws, neurons, *etc*.); which form links *L*_ij_(*c*_q_) in specific sub-set of conditions (*c*_q_). For this reason, we decided to call this strategy as ALMA (Assessing Links with Moving Averages) models. Speck-Planche and Cordeiro reported different multi-target or multi-output models using the same type of ALMA models [44,45,46].

In the specific area of neurodegenerative diseases, almost all these datasets includes also large sub-sets of assays involving potential neuroprotective drugs, targets, as well as drug-target and/or target–target interactions. The database NeuroDNet has interactive tools to create interaction networks for twelve neurodegenerative diseases. According to Vasaikar *et al*. [47], it is the first of its kind, which enables the construction and analysis of neurodegenerative diseases through protein interaction networks, regulatory networks and Boolean networks. In the case of neuroprotective compounds, some authors have reported multi-target ALMA models. García *et al*. used topological descriptors for a large series of 3370 active/non-active compounds to fit a classification function that can predict links *L*_ij_ (interactions) of heterogeneous series of GSK inhibitors compounds with different neurological targets relevant to Alzheimer’s disease and parasite species. Speck-Planche *et al*. [48], developed a multi-target model using a large and heterogeneous database of inhibitors against five proteins associated with Alzheimer’s disease. The model correctly classified more than 90% of active and inactive compounds in the treatment of Alzheimer’s disease on both, training and prediction series. Several guidelines are offered in other paper to show how the use of fragment-based descriptors can be determinant for the design of multi-target inhibitors of proteins associated with Alzheimer’s disease [49].

In a recent work, we used the method TOPS-MODE (TM) [50] to calculate the structural parameters of drugs. The model correctly classified 4393 out of 4915 total cases with Specificity (Sp), Accuracy (Ac), and Sensitivity (Sn), of 80%–98%. We also used the method TM to develop one ALMA [51] model useful for the prediction of neuroprotective drugs. This dataset includes Multi-output assay endpoints of 2217 compounds for at least one out of 338 assays, with 148 molecular or cellular targets, and 35 types of activity measures in 11 model organisms (including human). In a third work [52], we introduced another ALMA model for neurotoxicity/neuroprotective effects of drugs based on the method MI. First, we used MI to calculate molecular descriptors of the type of stochastic spectral moments of all compounds. Next, we found a model that classified correctly 2955/3548 total cases on training and validation series with Ac, Sn, and Sp > 80%. Each data point (>8000) contains the values of 37 possible measures of activity, 493 assays, 169 molecular or cellular targets, and 11 different organisms (including human) for a given compound. The model has shown excellent results also in computational simulations of high-throughput screening experiments, with Ac = 90.6% for 4671 positive cases. Both models are able to predict the links *L*_ij_(*c*_q_) between i^th^ drugs and j^th^ targets according to the assay *a*_q_. However, we do not carried out a formal construction and a comparison of the drug-target networks for the CHEMBL data in previous papers. In any case, despite the high versatility of entropy measures to codify structural information, there is no report of a multi-target model for drug–target interactions for compounds with neuroprotective/neurotoxic effect. In this work, we report the first multi-target, multi-output, and multi-scale ALMA model for CHEMBL data of neuroprotective/neurotoxic effect of drugs. Then, we construct and compare for the first time three Multi-output assay complex networks for these CHEMBL dataset using the two previous models and the model reported in this work. From there, we reported by the first time the synthesis, characterization, and experimental assays of a new series of rasagiline carbamate derivatives not reported in previous works. We carried out three different experimental tests: assay (1) in absence of neurotoxic agents; (2) in the presence of glutamate; and (3) in the presence of H_2_O_2_. Finally, we used the new entropy model to predict possible outcomes for these compounds in a high number of pharmacological tests not carried out experimentally. The results presented here show the high potential of entropy parameters of chemical information for the design of neuroprotective drugs, the construction of complex bio-molecular networks, and the potential of ALMA models for multi-target, multi-output, and multi-scale modeling.

## 2. Results and Discussion

### 2.1. Development of New Model for Prediction of Drug–Target Networks

#### 2.1.1. Model Training and Validation

We report a model to predicting when the i^th^ compound may present a high (*L*_ij_(*c*_q_) = 1) or not (*L*_ij_(*c*_q_) = 0) value of the experimental parameter used to characterize interaction with a molecular or cellular target involved in a neuroprotective/neurodegenerative process. The output *S*_ij_(*c*_q_) of our multi-output model depend on both chemical structure of the i^th^ drug *d*_i_ and the set of conditions selected to perform the biological assay (*c*_q_) including the j^th^ target, of course. In consonance, the ALMA model should predict different probabilities if we change the organisms (*c*_1_), the biological assays (*c*_2_), the molecular/cellular target (*c*_3_), or the standard experimental parameter measured (*c*_4_), for the same compound [53].The best ALMA-entropy model found in this work was:

(1) $$\begin{array}{l} S_{ij}\left(c_{q}\right)=\;1.1396-0.4039\cdot p(c_{l})\theta_{1}^{i}+0.1993\cdot \Delta \theta_{1}^{i}\left(s_{x}\right)+0.4349\cdot \Delta \theta_{1}^{i}\left(a_{u}\right)\\ -0.0202\cdot \Delta \theta_{1}^{i}\left(o_{t}\right)-0.0017\cdot \Delta \theta_{1}^{i}\left(t_{e}\right)\\ N=2661\;R_{c}\;=\;0.72\;\chi^{2}=1913.007\;p<0.005 \end{array}$$

The statistical parameters for the above equation in training are: Number of cases used to train the model (*N*), Canonical Regression Coefficient (*R*_c_), Chi-square (χ^2^), and *p*-level [54]. The probability cut-off for this Linear Discriminant Analysis (LDA) model is ^i^*p*_1_(*c*_q_) > 0.5 ≥ *L*_ij_(*c*_q_) = 1. It means that the drug *d*_i_ predicted by the model, with probability *p* > 0.5, is expected to give a positive outcome in the q^th^ assays carry out under the given set of conditions *c*_q_. This ALMA-entropy model presents excellent performance in both training and external validation series with Sn, Sp, and Ac > 80% (see Table 1). Values higher than 75% are acceptable for LDA-QSAR models, according to previous reports [55,56,57,58,59].

The first term in the equation, quantify both the quality of the input data *p*(*c*_l_) and the information θ^i^_5_ about the structure of the drug (see material and methods and previous works [51]). We can expand the Box–Jenkins MA terms in the ALMA equation in order to clearly depict all the parameters involved:

(2) $$\begin{array}{l} S_{ij}\left(c_{q}\right)=\;1.139556-0.403994\cdot p_{1}\left(s_{x}\right)\cdot \theta_{5}^{i}\\ +0.199322\cdot \left[\theta_{5}^{i}\left(s_{x}\right)-p_{1}\left(s_{x}\right)\cdot \langle \theta_{5}\left(s_{x}\right)\rangle \right]\\ +0.434889\cdot \left[\theta_{5}^{i}\left(a_{u}\right)-p_{1}\left(a_{u}\right)\cdot \langle \theta_{5}\left(a_{u}\right)\rangle \right]\\ -0.020189\cdot \left[\theta_{5}^{i}\left(o_{t}\right)-p_{1}\left(o_{t}\right)\cdot \langle \theta_{5}\left(o_{t}\right)\rangle \right]\\ -0.001660\cdot \left[\theta_{5}^{i}\left(t_{e}\right)-p_{1}\left(t_{e}\right)\cdot \langle \theta_{5}\left(t_{e}\right)\rangle \right]\\ N=2661\;R_{c}\;=\;0.72\;\chi^{2}=1913.007\;p<0.005 \end{array}$$

After inspection of this equation, we can see that the ALMA model can predict for the same compound different scores for different experimental parameters, targets, assays, or even different organisms. In Table 2 we illustrate the values of probability of drug–target interaction *p*_ij_(*c*_q_) predicted with the previous model, for several examples of known drugs or new promising compounds. These are the probabilities with which the i^th^ compound interact with the j^th^ drug under the assay conditions *c*_q_. This is equivalent to *p*_ij_(*c*_q_) > 0.5 ≥ *L*_ij_(*c*_q_)_pred_ = 1. However, online supplementary material files contain a complete list with many examples of positive and control cases.

**Table 1:** Results of Assessing Links with Moving Averages (ALMA) models for entropy measures *vs*. different spectral moments.

Descriptor	Sub-Set	Stat. ^a^	%	Groups	*C*_i_(*m*_j_)_pred_ = 1	*C*_i_(*m*_j_)_pred_ = 0	Reference
MI-Entropy	Train	Sp	79.0	*L*_ij_(*C*_q_)_obs_ = 1	**1092**	290	This work
		Sn	91.5	*L*_ij_(*C*_q_)_obs_ = 0	412	**4438**	
		Ac	88.7	Total			
	CV	Sp	81.3	*L*_ij_(*C*_q_)_obs_ = 1	**379**	87	
		Sn	92.6	*L*_ij_(*C*_q_)_obs_ = 0	119	**1492**	
		Ac	90.1	Total			
MI spectral moments	Train	Sp	84.6	*L*_ij_(*C*_q_)_obs_ = 1	**1172**	214	[52]
		Sn	82.4	*L*_ij_(*C*_q_)_obs_ = 0	224	**1051**	
		Ac	83.5	Total			
	CV	Sp	83.3	*L*_ij_(*C*_q_)_obs_ = 1	**385**	77	
		Sn	81.6	*L*_ij_(*C*_q_)_obs_ = 0	78	**347**	
		Ac	82.5	Total			
TM spectral moments	Train	Sp	81.3	*L*_ij_(*C*_q_)_obs_ = 1	**1533**	352	[51]
		Sn	98.0	*L*_ij_(*C*_q_)_obs_ = 0	36	**1762**	
		Ac	89.5	Total			
	CV	Sp	81.0	*L*_ij_(*C*_q_)_obs_ = 1	**513**	120	
		Sn	97.7	*L*_ij_(*C*_q_)_obs_ = 0	14	**585**	
		Ac	89.1	Total			

MI, MARCH-INSIDE; ^a^ Sensitivity = Sn = Positive **Correct**/Positive Total; Specificity = Sp = Negative **Correct**/Negative Total; Accuracy = Ac = Total **Correct**/Total; TM, TOPS-MODE.

**Table 2:** Examples predicted with the model.

Compound (i)	*p*_ij_(*c*_q_)	Assay ID	Measure (Units)	Organism	Target Protein
Arecoline	0.94	796814	Efficiency (%)	rno	Muscarinic acetylcholine receptor
Bipinnatin-A	1.00	751272	Inhibition (%)	mmu	Acetylcholine receptor protein β chain
Carachol	0.99	796814	Efficiency (%)	rno	Muscarinic acetylcholine receptor
Caulophylline	0.96	838016	EC_50_ (nM)	hsa	Neuronal acetylcholine receptor; α4/β2
Citalopram	0.99	740208	*K*_i_ (nM)	mmu	Dopamine transporter
Condelphine	1.00	748943	−Log(IC_50_) (nM)	rno	Neuronal acetylcholine receptor protein α-7 subunit
Delcorine	1.00	748943	−Log(IC_50_) (nM)	rno	Neuronal acetylcholine receptor protein α-7 subunit
Delsoline	1.00	748943	−Log(IC_50_) (nM)	rno	Neuronal acetylcholine receptor protein α-7 subunit
Desipramine	0.99	797692	−Log(IC_50_) (nM)	rno	Norepinephrine transporter
Elatine	1.00	748943	−Log(IC_50_) (nM)	rno	Neuronal acetylcholine receptor protein α-7 subunit
Emopamil	1.00	817225	−Log(IC_50_) (nM)	rno	Voltage-gated R-type calcium channel α-1E subunit
Epibatidine	0.94	838016	EC_50_ (nM)	hsa	Neuronal acetylcholine receptor; α4/β2
Epibatidine	0.19	825420	Efficacy (%)	hsa	Neuronal acetylcholine receptor; α4/β2
Femoxetine	0.99	740206	*K*_i_ (nM)	mmu	Dopamine transporter
Femoxetine	0.99	740207	*K*_i_ (nM)	mmu	Norepinephrine transporter
Femoxetine	0.99	740208	*K*_i_ (nM)	mmu	Dopamine transporter
Fisetin	0.05	1027709	%max (%)	mmu	HT22 cells
Fluoxetine	0.99	740207	*K*_i_ (nM)	mmu	Norepinephrine transporter
Fluoxetine	0.99	740208	*K*_i_ (nM)	mmu	Dopamine transporter
Imipramine	0.99	740206	*K*_i_ (nM)	mmu	Dopamine transporter
Imipramine	0.99	740207	*K*_i_ (nM)	mmu	Norepinephrine transporter
Imipramine	0.99	740208	*K*_i_ (nM)	mmu	Dopamine transporter
Inuline	1.00	748943	−Log(IC_50_) (nM)	rno	Neuronal acetylcholine receptor protein α-7 subunit
Karacoline	1.00	748943	−Log(IC_50_) (nM)	rno	Neuronal acetylcholine receptor protein α-7 subunit
l-Arginine	0.99	755144	Activity (nM)	hsa	Nitric-oxide synthase, brain
l-NIL	0.59	752266	−Log(IC_50_) (nM)	hsa	Nitric-oxide synthase, brain
l-NMMA	0.99	876477	−Log(IC_50_) (nM)	hsa	Nitric-oxide synthase, brain
l-NNA	0.98	752385	−Log(IC_50_) (nM)	hsa	Nitric-oxide synthase, brain
l-NNA	0.86	752276	*K*_i_ (nM)	hsa	Nitric-oxide synthase, brain
LY-379268	0.99	714803	Activity (nM)	hsa	Metabotropic glutamate receptor 4
LY-379268	0.99	877752	Activity (nM)	hsa	Metabotropic glutamate receptor 2
LY-379268	0.99	718128	Activity (nM)	hsa	Metabotropic glutamate receptor 6
LY-389795	0.99	718128	Activity (nM)	hsa	Metabotropic glutamate receptor 6
LY-389795	0.98	715721	Activity (nM)	hsa	Metabotropic glutamate receptor 5
LY-389795	0.97	714446	Activity (nM)	hsa	Metabotropic glutamate receptor 3
Lycoctonine	1.00	748943	−Log(IC_50_) (nM)	rno	Neuronal acetylcholine receptor protein α-7 subunit
M826	1.00	841780	*K*_i_ (nM)	hsa	Caspase-3
M827	1.00	841780	*K*_i_ (nM)	hsa	Caspase-3
Methyllycaconitine	1.00	750084	*K*_i_ (nM)	rno	Neuronal acetylcholine receptor protein α-10 subunit
NBQX	0.99	641893	−Log(IC_50_) (nM)	rno	Glutamate receptor ionotropic, AMPA 2
NBQX	0.99	641893	−Log(IC_50_) (nM)	rno	Glutamate receptor ionotropic, AMPA 4
NBQX	0.99	641893	−Log(IC_50_) (nM)	rno	Glutamate receptor ionotropic, AMPA 3
NBQX	0.99	641893	−Log(IC_50_) (nM)	mmu	Glutamate receptor ionotropic, AMPA 1
Nipecotic acid	0.28	785010	−Log(IC_50_) (nM)	rno	GABA transporter 1
Nipecotic acid	0.28	785010	−Log(IC_50_) (nM)	rno	GABA transporter 2
Nipecotic acid	0.28	785010	−Log(IC_50_) (nM)	rno	GABA transporter 3
Nipecotic acid	0.28	785010	−Log(IC_50_) (nM)	rno	Betaine transporter
NOHA	0.04	755137	NO formation (%)	rno	Nitric-oxide synthase, brain
Norepinephrine	0.98	780755	Concentration (% dose·g^−1^)	rno	
Nudicauline	1.00	748943	−Log(IC_50_) (nM)	rno	Neuronal acetylcholine receptor protein α-7 subunit
Omega nitro-arginine	0.99	752258	*K*_i_ (nM)	hsa	Nitric-oxide synthase, brain
Oxotremorine	0.84	798083	pD2	rno	Muscarinic acetylcholine receptor M1
Paroxetine	1.00	740206	*K*_i_ (nM)	mmu	Dopamine transporter
RedAm-Ethyl	0.33	840782	Selectivity	hsa	Nitric-oxide synthase, endothelial
RedAm-Ethyl	0.28	840782	Selectivity	hsa	Nitric-oxide synthase, brain
Resveratrol	0.99	1613870	EC_50_ (nM)	hsa	Nuclear factor NF-κB p105 subunit
Resveratrol	0.99	1613870	EC_50_ (nM)	hsa	Nuclear factor NF-κB p65 subunit
Stemofoline	1.00	936299	EC_50_ (nM)	hvi	Nicotinic acetylcholine receptor α1 subunit
Thiocytisine	0.51	857972	Log *K*_i_	rno	Neuronal acetylcholine receptor; α4/β2

rno, *Rattus norvegicus* (Rat); mmu, *Mus musculus* (Mouse); hsa, *Homo sapiens* (Human); and hvi, *Heliothis virescens*.

The Table 2 shows predictions of the same drug in different sets of conditions of assay *c*_q_, including different targets, organisms, or assays. Therefore, we only have to substitute in the equation the value of θ^i^_5_ of the compound and the respective values *p*_1_(*c*_q_)·<θ^i^_5_(*c*_q_)> for the MA operators of each condition. In the Table 3 we depict many examples of values of MA operators *p*_1_(*c*_q_)·<θ^i^_5_(*c*_q_)> for different conditions.

#### 2.1.2. Comparison with Other ALMA Models

An interesting exercise is the comparison of the present model and the network predicted with outcomes obtained with other methods. Until the best of our knowledge, there are only two similar models. Both models make use of the spectral moments of a molecular matrix as input variables (*D*_i_) to quantify the molecular structure of drugs. The first model [51] applies spectral moments µ_k_ of order k^th^ of the bond adjacency matrix (^1^**B**) calculated with the TM approach. The equation of this model is the following:

(3) $$\begin{array}{l} S_{ij}\left(c_{q}\right)=\;-7.01\cdot 10^{-4}\cdot p\left(c_{j}\right)\cdot \mu_{5}^{i}-7.84\cdot 10^{-4}\cdot \Delta \mu_{5}^{i}\left(s\right)-2.93\cdot 10^{-4}\cdot \Delta \mu_{5}^{i}\left(a\right)\\ +1.16\cdot 10^{-4}\cdot \Delta \mu_{5}^{i}\left(o\right)+2.84\cdot 10^{-4}\cdot \Delta \mu_{5}^{i}\left(t\right)+4.198684\\ N=3683\;R_{c}\;=\;0.7\;p<0.005 \end{array}$$

The second model [52] employs as input the π^i^_k_ values of the Markov matrix (^1^**Π**) of atom–atom electron delocalization calculated with the software MI. In the TM method, we weighted the edges of the molecular graph with standard distances of chemical bonds whereas the MI algorithm employs atom standard electronegativities to weighting the nodes of molecular graph. The equation of the second model is:

(4) $$\begin{array}{l} S_{ij}\left(c_{q}\right)=\;1.139556-0.403994\cdot p\left(c_{j}\right)\cdot \pi_{5}^{i}+0.199322\cdot \Delta \pi_{5}^{i}\left(s_{x}\right)+0.434889\cdot \Delta \pi_{5}^{i}\left(a_{u}\right)\\ -0.020189\cdot \Delta \pi_{5}^{i}\left(o_{t}\right)-0.001660\cdot \Delta \pi_{1}^{i}\left(t_{e}\right)\\ N=2661\;R_{c}\;=\;0.72\;\chi^{2}=1913.007\;p<0.005 \end{array}$$

In both cases, as well as in the present ALMA-entropy model, we used MA terms to quantify the deviations of the structure of one compound from sub-sets of compounds with a positive outcome in different conditions *c*_q_. The three methods showed excellent values of Ac, Sp, and Sn on both training and validation series (see Table 1). Apparently, the TM model shows better values of these parameters but we have to take into consideration the differences in the complexity of the data sets used to train and validate these models. The TM-spectral moment model is able to classify correctly 83%–82% of 4915 cases in total (on training and validation series respectively). The MI-spectral moment model is able to classify correctly 89%–92% of 3598 cases. Notably, the MI-entropy model is able to classify correctly 89%–92% of 8309 cases. Consequently, the statistics for the present model refer to a dataset with more than twice the number of data points present in previous models.

**Table 3:** Examples of multi-scale, multi-target, or multi-output MA values for different targets, measures, and organisms.

Experimental Measure (units)	Statistics			*p*_1_(*c*_j_)·<θ_k_(*c*_q_)>					Experimental Measure (units)	Statistics			*p*_1_(*c*_j_)·<θ_k_(*c*_q_)>				
	*n*(*s*_x_)	*n*_1_(*s*_x_)	*p*_1_(*s*_x_)	1	2	3	4	5		*n*(*s*_x_)	*n*_1_(*s*_x_)	*p*_1_(*s*_x_)	1	2	3	4	5
−Log(IC_50_) (nM)	2438	2148	0.88	2.03	2.08	2.04	2.04	2.03	ED_50_ (μg·kg^−1^)	19	14	0.74	1.58	1.6	1.59	1.59	1.59
EC_50_ (nM)	2149	1975	0.92	1.87	1.91	1.89	1.89	1.88	ED_50_ (nM)	18	14	0.78	2.14	2.17	2.14	2.14	2.13
*K*_i_ (nM)	1501	1418	0.94	2.01	2.06	2.03	2.02	2.01	NO formation (%)	18	6	0.33	0.63	0.64	0.63	0.63	0.63
Selectivity	486	102	0.21	0.5	0.51	0.51	0.51	0.51	Efficiency (%)	14	11	0.79	1.58	1.61	1.6	1.6	1.59
Dopamine release (%)	299	130	0.43	0.89	0.91	0.89	0.89	0.88	*K*_up_ (mL·min^−1^·g^−1^)	13	5	0.38	0.75	0.76	0.76	0.76	0.76
Activity (%)	222	105	0.47	1.22	1.24	1.23	1.23	1.22	Conc. (%·dose·g^−1^)	12	7	0.58	1.14	1.15	1.14	1.14	1.14
Inhibition (%)	193	93	0.48	0.99	1	0.99	0.99	0.98	Efficacy (%)	12	6	0.5	0.58	0.58	0.58	0.59	0.59
Selectivity ratio	166	61	0.37	0.94	0.95	0.93	0.93	0.92	Ratio *K*_i_	12	2	0.17	0.38	0.39	0.39	0.39	0.39
Log *K*_i_	124	72	0.58	0.96	0.97	0.96	0.96	0.96	MTT reduction (%)	11	4	0.36	0.58	0.57	0.57	0.56	0.56
Ratio	108	31	0.29	0.66	0.67	0.66	0.66	0.65	Relative potency	11	4	0.36	0.93	0.94	0.92	0.92	0.91
Activity (nM)	98	93	0.95	1.74	1.77	1.75	1.75	1.74	ED_50_ (μg·mL^−1^)	10	4	0.4	0.99	1.02	0.99	0.99	0.98
PCMA antagonism	84	26	0.31	0.51	0.51	0.51	0.52	0.52	Activity	8	5	0.63	1.98	2.01	1.99	1.99	1.99
−Log(IC_50_)	56	17	0.3	0.56	0.58	0.57	0.57	0.57	Damage score	8	2	0.25	0.5	0.51	0.5	0.49	0.49
Ratio (nM)	56	32	0.57	1.1	1.12	1.11	1.1	1.1	Mean response	8	5	0.63	1.44	1.48	1.46	1.46	1.46
*n*NOS activity (%)	36	25	0.69	1.69	1.73	1.7	1.69	1.68	Survived (%)	8	5	0.63	1.04	1.04	1.04	1.04	1.04
%max (%)	20	4	0.2	0.55	0.56	0.55	0.55	0.54	Rescued neurons (%)	5	2	0.4	0.59	0.6	0.61	0.62	0.63
Organism	*n*(*o*_j_)	*n*_1_(*o*_j_)	*p*_1_(*o*_j_)	1	2	3	4	5	Organism	*n*(*o*_j_)	*n*_1_(*o*_j_)	*p*_1_(*o*_j_)	1	2	3	4	5
*R*. *norvegicus*	2852	1998	0.7	1.51	1.54	1.52	1.52	1.51	*B*. *taurus*	77	21	0.27	0.63	0.63	0.63	0.63	0.63
*H*. *sapiens*	4854	4090	0.84	1.82	1.86	1.83	1.83	1.82	*C*. *porcellus*	20	16	0.8	1.35	1.36	1.35	1.35	1.35
*F*. *catus*	10	7	0.7	1.66	1.7	1.68	1.67	1.66	*H*. *virescens*	5	5	1	2.78	2.83	2.78	2.78	2.76
*M*. *musculus*	241	173	0.72	1.5	1.53	1.51	1.51	1.51	*M*. *domestica*	15	15	1	1.62	1.66	1.67	1.68	1.68
*T*. *californica*	19	11	0.58	1.34	1.37	1.35	1.35	1.34	*C*. *elegans*	2	1	0.5	1.28	1.31	1.28	1.27	1.26
*Gerbillinae*	8	2	0.25	0.5	0.51	0.5	0.49	0.49	*D*. *melanogaster*	2	1	0.5	1.28	1.31	1.28	1.27	1.26
Protein ACC.	*n*(*t*_j_)	*n*_1_(*t*_j_)	*p*_1_(*t*_j_)	1	2	3	4	5	Name								
Q9UGM1	403	254	0.63	1.34	1.36	1.34	1.34	1.34	Neuronal acetylcholine receptor protein α-9 subunit								
Q62645	77	21	0.27	0.63	0.63	0.63	0.63	0.63	Glutamate (NMDA) receptor subunit ε 4								
P35228	128	32	0.25	0.53	0.54	0.53	0.53	0.53	Nitric oxide synthase, inducible								
P29476	859	562	0.65	1.30	1.32	1.30	1.30	1.30	NOS, brain								
P29474	88	18	0.20	0.50	0.51	0.50	0.50	0.50	NOS, endothelial								
P19838	1000	923	0.92	1.88	1.91	1.89	1.89	1.88	Nuclear factor NF-κB p105 subunit								
P12392	104	90	0.87	1.82	1.87	1.84	1.84	1.82	Neuronal acetylcholine receptor protein β-4 subunit								
P12390	79	66	0.84	1.78	1.83	1.80	1.80	1.79	Neuronal acetylcholine receptor protein β-2 subunit								
P12389	37	31	0.84	1.97	2.04	2.01	2.01	2.00	Neuronal acetylcholine receptor protein α-2 subunit								
P09483	29	28	0.97	1.98	2.02	2.00	1.99	1.98	Neuronal acetylcholine receptor protein α-4 subunit								
Assay ID	*n*(*c*_j_)	*n*_1_(*c*_j_)	*p*_1_(*c*_j_)_j_	1	2	3	4	5	Details								
1613870	2000	1846	0.92	1.88	1.91	1.89	1.89	1.88	Expression of NF-κB in human neuronal cells								
832611	646	646	1.00	2.31	2.37	2.32	2.30	2.28	Inhibition of [3*H*]EBOB binding to γ-aminobutyric acid GABA–AR								
842916	390	390	1.00	2.12	2.16	2.13	2.12	2.11	[Ca^2+^] influx in neonatal rat spinal sensory neuronal culture								
792863	299	130	0.43	0.89	0.91	0.89	0.89	0.88	Binding of norditerpenoid alkaloids at neuronal α7 nicotinic AChR								
899883	114	99	0.87	1.46	1.48	1.46	1.46	1.45	Membrane potential in K-177 cells with ACh central neuronal receptor								
1041434	74	17	0.22	0.56	0.57	0.56	0.56	0.56	mGluR-6 influence in c-AMP formation in rat nonneuronal cells								
829510	50	50	1.00	2.93	2.97	2.93	2.91	2.89	Inhibition of glutamate induced neuronal death								
829508	50	50	1.00	2.93	2.97	2.93	2.91	2.89	Inhibition human caspase-1 in neuronal precursor (NT2) cells								
829511	50	50	1.00	2.93	2.97	2.93	2.91	2.89	Inhibition human caspase-8 in neuronal precursor (NT2) cells								
1814959	11	11	1.00	2.95	3.00	2.96	2.94	2.92	Blocking permeability of the neuronal Na^+^ in rat striatum slices								

#### 2.1.3. Construction of Drug–Target Networks with ALMA Models

ALMA models may be useful both (1) for computational or virtual High-Throughput Screening (HTS) screening of large databases like CHEMBL and/or (2) for construction of drug–target networks. All the results, discussed in previous section, indicate that many compounds may act as multi-target drugs with non-linear or indirect effect (orthosteric and/or allosteric) over different targets in different pathways. In a recent special issue edited by Csermely, Nussinov, and Szilágyi [60], different research groups discussed about this topic and related concepts such as allo-networks. In one of these papers, Mueller *et al*. [61] have developed a computational model for the HTS of drugs with action over mGluR5; which represent a promising strategy for the treatment of schizophrenia. Considering the relevance of allotropy for these and other receptors for our study, and all previous comments about allo-network drugs, we decided to use our model to construct a drug-target network. The interest in doing so is that this type of network-based tools may be applied for the discovery of new drugs, including perhaps allo-network drugs [40,60,62].

Considering these points, we constructed here by the first time a drug–target network with CHEMBL experimental outcomes of multiplex assays of neuroprotective effects of drugs with the same dataset used in the previous section. This is probably the first drug–target network representation of the interaction of neuroprotective compounds with cellular or protein targets; many of them susceptible to allosteric modulators. In this directed network, we used three classes of nodes, drugs (*d*_i_), targets (*t*_j_), and pharmacological assays (*a*_q_). They are connected by only three classes of arcs (directed links) drug ≥ (*d*_i_ ≥ *t*_j_), drug ≥ assay (*d*_i_ ≥ *a*_q_), and target-assay (*t*_j_ ≥ *a*_q_). Other types of relationships were not considered. The observed drug–target network was constructed with the input dependent variable *L*_ij_(*c*_q_). In consequence, if CHEMBL reports the case of drug *d*_i_ that causes an strong biological response (*L*_ij_(*c*_q_) = 1) in one biological experiment carry out under the conditions *c*_q_ = (*t*_j_, *a*_q_), we have to draw in the network the path *d*_i_ ≥ *t*_j_ ≥ *a*_q_. We omitted here the representation of nodes for the type of experimental measure and the organism that express the target. This avoids very highly connected nodes that may cause a strong distortion in network topology and mask or hidden the relevance of important drugs or targets.

The observed network constructed with the dataset published in the previous work has 968 nodes = 721 drugs + 72 targets + 175 pharmacological assays for neuroprotective effects. We apply, also, the software MI to quantify the structural information of the drug–target networks. In so doing, we calculated the Shannon entropy (Sh), as well as δ = node degrees for the nodes (drugs, targets, and assays) in the network, see Table 4. Please note that the Sh entropy values for the nodes in the drug–target network (supra-molecular structural level) are different from the θ_k_ entropy values use to quantify the information about the structure of the drug (molecular structural level). Actually, we do not use a classic Shannon entropy (H) but a first-order Markov–Shannon entropy [25].

After a first inspection, we can observe that the degree of a node (δ) in the network has average values of δ = 4.8 ≈ 5 for all nodes, δ = 4.8 ≈ 5 for drugs, and δ = 4.3 ≈ 4 for assays. It means that, on average, each drug interacts with five targets and we can measure this interaction with approximately four assays. It is easy to realize that the higher δ for targets may be determined in part by their position in the network. For each link of drug or assay node, we have two interactions for the target *d*_i_ ≥ *t*_j_ and *t*_j_ ≥ *a*_q_). As a result, we can decompose the δ into δ = δ_in_ + δ_out_ = node degree = in-degree + out-degree [63]. For this reason, we carried out all calculations eliminating the direction of arcs. In so doing, we considered them as symmetric links to avoid this “over-booking” of target nodes. Consequently, the average is δ = 6.1 ≈ 6 for targets, a value still higher, but closer to 5 than to 8–10, the double is expected.

**Table 4:** Topological properties of CHEMBL complex networks predicted with ALMA-entropy models.

Network	Node Type	*n*	Sh_1_^a^	δ	δ_in_	δ_out_
Observed	Total	2450	0.00428	7	3	3
	Compounds	2103	0.00413	6	3	3
	Assays	211	0.00575	6	3	3
	Rat proteins	54	0.00291	7	4	3
	Human proteins	70	0.00568	21	18	3
1	Total	2508	0.00438	7	3	3
	Compounds	2208	0.00446	6	3	3
	Assays	183	0.00468	15	11	4
	Rat proteins	40	0.00279	6	3	3
	Human proteins	67	0.00210	5	1	3
2	Total	2511	0.00428	7	3	3
	Compounds	2209	0.00445	6	3	3
	Assays	184	0.00464	15	11	4
	Rat proteins	40	0.00266	6	3	3
	Human proteins	68	0.00209	4	1	3
3	Total	2511	0.0044	7	3	3
	Compounds	2209	0.00445	6	3	3
	Assays	184	0.00464	15	11	4
	Rat proteins	40	0.00266	6	3	3
	Human proteins	68	0.00209	4	1	3
4	Total	2491	0.0046	7	3	3
	Compounds	2209	0.00471	6	3	3
	Assays	184	0.00449	14	11	4
	Rat proteins	40	0.00251	6	2	3
	Human proteins	68	0.00209	4	1	3
5	Total	2491	0.0046	7	3	3
	Compounds	2209	0.00471	6	3	3
	Assays	184	0.00449	14	11	4
	Rat proteins	40	0.00251	6	2	3
	Human proteins	68	0.00209	4	1	3

^a^ δ = δ_in_ + δ_out_ = node degree = in-degree + out-degree, Sh_1_ = Shannon entropy of Markov chain (measure of information).

**Figure 1:**
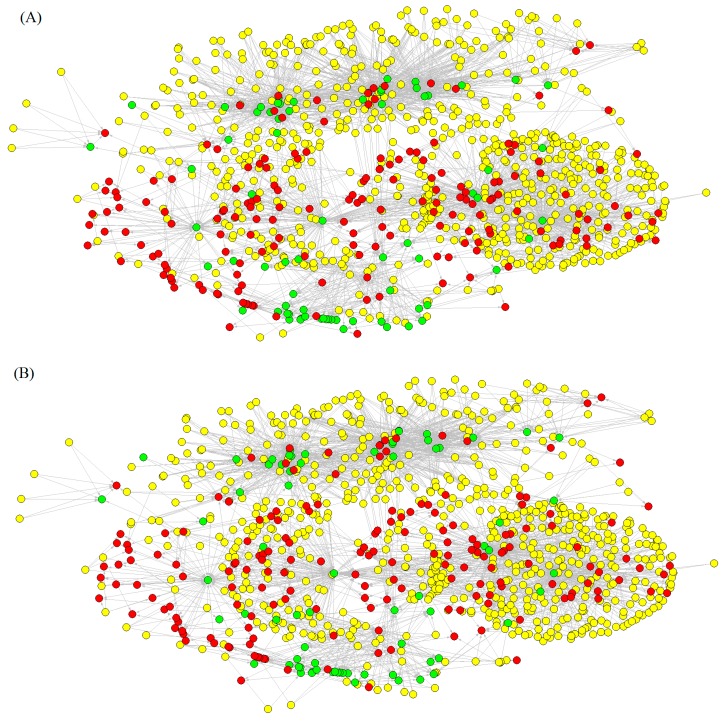
Multitarget, Multiscale, and Multi-output networks, of CHEMBL sub-set of neuroprotection related drugs (yellow), targets (red), and pharmacological assays (green) Observed (**A**) *vs*. Predicted (**B**).

In a second stage, we use our model to reconstructing/predicting the same network, based on the probability p(m_j_) outputs of the model. Two nodes are connected when the probability predicted by the model is p(c_q_) > 0.5, it means that p(d_i_, t_j_), or p(d_i_, a_q_), or p(t_j_, a_q_) are >0.5, for different pairs of links. We can perceive that the values of the drug–target network predicted by the model are very similar to those of the observed network. Consequently, we can conclude that the model is efficient not only in the overall prediction of links in the network (high Ac, Sp, and Sn, see Table 1) but in the reconstruction of topological patterns. For instance, from information theory we can deduce that the uncertainty of links is similar in both networks because Shannon entropy calculated for all links is Sh_obs_ = 0.005 − 0.007 ≈ Sh_pred_ = 0.004 − 0.006. In Figure 1, we represented the Observed (A) *vs*. Predicted (B) complex networks.

### 2.2. Experimental and Theoretical Study of New Compounds

#### 2.2.1. Synthesis and Experimental Assay of New 1,2-Rasagiline Derivatives

**Figure 2:**
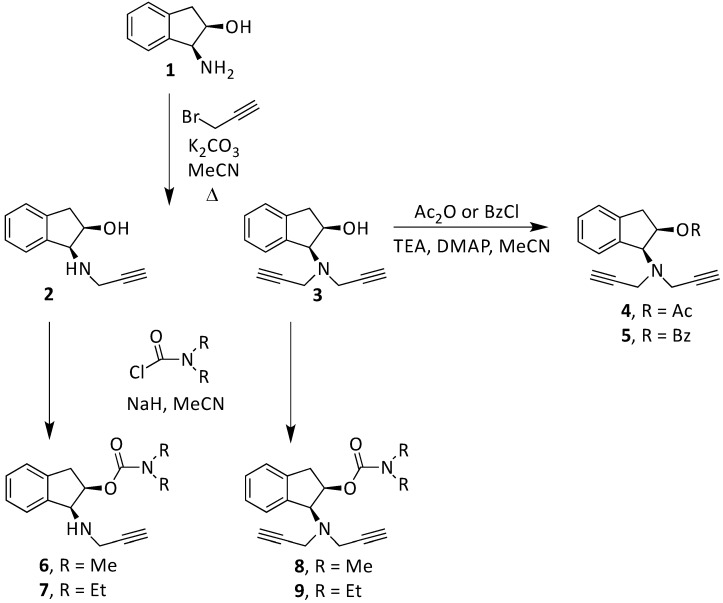
Synthesis of compounds **2**–**9**.

The compounds **2**, **3**, **4**, **5**, **6**, **7**, **8**, and **9** were synthesized according to the strategy given in Figure 2. As shown in this scheme, they were synthesized from the aminoalcohol **1** [(1*R*,2*S*)-(+)-1-amino-2-indanol], a commercial product. The alkylation of **1** with propargyl bromide and potassium carbonate in hot acetonitrile provided, in a global yield of 92%, a mixture of the corresponding mono- and dipropargylated derivatives (**2** and **3**), which were separated by flash column chromatography using hexane/EtOAc (3:1) as eluent. Compound **3** was converted to the corresponding acetate (**4**) and benzoate (**5**) by treatment with acetic anhydride or benzoyl chloride, Et_3_N and catalytic amounts of 4-dimethylaminopyridine (DMAP) in MeCN. The carbamate derivatives (**6**, **7**, **8**, and **9**) were synthesized, from the hidroxy mono- or dipropargylaminoindans (**2** and **3**), by reaction with the corresponding dialkylcarbamyl chloride in NaH and acetonitrile following the procedure described in the literature [64].

The new compounds synthesized in this work (**2**, **3**, **4**, **5**, **6**, **7**, **8**, and **9**) were subjected to an initial study to determinate its neuroprotective ability in both the presence and the absence of neurotoxic agents (ANA). The method of reduction of the 3-(4,5-dimethylthiazol-2-yl)-2,5-diphenyltetrazolium bromide (MTT) was used to ascertain the cell viability, given by the number of cells present in the culture. The ability of cells to reduce MTT is an indicator of the integrity of mitochondria, and its functional activity is interpreted as a measure of cell viability [65]. Three assays were conducted in a culture of motor cortex neurons of 19-day-old Sprague–Dawley rat embryos. All results are expressed as the mean ± S.E.M. [51,52] of at least three independent experiments (Table 5).

**Table 5:** Neuroprotective ability of the new 1,2-rasagiline derivatives.

Compound	Formula	% Neuro-Protection					
		% ANA ^a^	e.s.m.	Glutamate ^b^	e.s.m.	H_2_O_2_^c^	e.s.m.
**2**	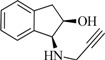	0.0	2.8	0.0	6.5	−2.8	1.2
**3**	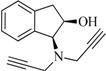	4.7	6.0	−0.2	1.6	−12.3	2.1
**4**	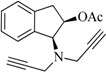	4.2	6.5	−8.1	4.9	−14.2	2.1
**5**	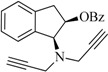	1.2	5.0	3.8	5.0	2.9	1.0
**6**	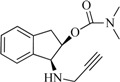	11.5	8.8	−4.0	5.5	−9.1	2.4
**7**	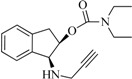	4.0	4.5	2.6	3.9	-6.1	1.1
**8**	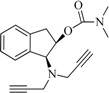	−1.7	6.9	−5.2	5.9	−8.9	1.9
**9**	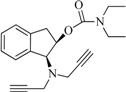	8.4	10.7	−5.2	2.3	−14.0	2.0

^a^ % protection (comp 5 µM), in the Absence of Neurotoxic Agents (ANA); ^b^ % protection (comp 5 µM) against Glutamate 100 µM; ^c^ % protection (comp 5 µM) against H_2_O_2_ 100 µM.

**Figure 3:**
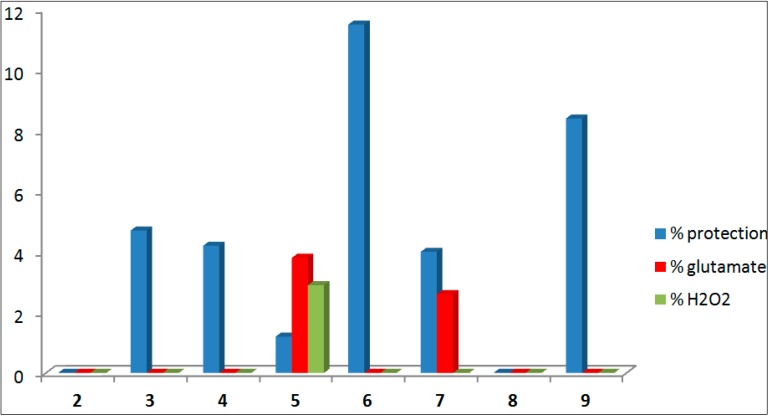
Results of the experimental assay of neuroprotective effect of the new compounds.

Firstly, we studied the ability to induce a neuroprotective effect in the absence of any neurotoxic stimulation. Secondly, we studied the neuroprotective effect in the presence of glutamate, a compound that causes a pathological process, in which neurons are damaged leading to apoptosis when its receptors, such as the NMDA and AMPA, are over-activated. Lastly, the ability of the compounds synthesized to protect from damage by H_2_O_2_, that causes neuronal death by oxidative stress, was analyzed. The results obtained allow to deduce the existence of a moderate neuroprotective effect in the absence of any toxic stimulus, presenting the best results type **6** and **9** carbamate derivatives, with values of 11.5% and 8.4%, respectively, followed by the compound **3**, **4**, and **7** with values slightly above 4% (see Figure 3).

#### 2.2.2. Using ALMA-Entropy Model to Predicting New Drugs in Other Assays

We used the ALMA-entropy model to predicting the more probable results for all the new rasagiline derivatives synthesized in this work, in >500 assays not carried out experimentally. When the molecular descriptors (entropy indices) of the new rasagiline derivatives were introduced in our model, we obtained the probable interaction with different targets. The model predicts that most of them could interact with the subunits A and B of the 5-hidroxy-tryptamine type 3 receptors (5-HT3Rs), see Table 6. These results seem to be consistent with the literature, since the antagonists of 5-HT3Rs have been related to neuroprotective properties *in vitro* and *in vivo* [66]. In fact, this could be a potential mechanism of neuroprotection added to several described mechanisms for rasagiline derivatives [67,68]. Rasagiline is also known for promoting serotoninergic activity by other ways, which is a clinically relevant fact in certain circumstances [69]. All in one highlights the intricate relationships of these drugs with the 5-hidroxy-tryptamine (serotonine) system.

**Table 6:** Some predictive results for interaction between compound **6** with 5HT3Rs and other targets.

*S*_i_(*c*_j_)	Meassure	Assay ID	Target ID	Target ^a^	Neurotoxic Agent
2.097	pA2	617971	1899	5HT3aR	ANA
2.097	pA2	617969	1899	5HT3aR	ANA
2.097	pA2	617971	3895	5HT3bR	ANA
2.097	pA2	617969	3895	5HT3bR	ANA
1.78	Selectivity	848737	3568	bNOS	H_2_O_2_
1.78	Selectivity	840777	3568	bNOS	H_2_O_2_
1.78	Selectivity	755901	3568	bNOS	H_2_O_2_
1.17	Activity (%)	866501	2586	nAChRβ-3	H_2_O_2_
0.42	pIC_50_ (nM)	710048	3772	mGluR1	Glu

^a^ nAChRβ-3 = neuronal acethyl-choline receptor β3, mGluR1 = metabotropic glutamate receptor type 1.

In any case, we need to analyze these results with caution. In our previous works [51,52], we predicted with new models and confirmed experimentally that some rasagiline derivatives (similar to the derivatives studied in this work) presented activity over glutamate receptors (GluRs) pathway. In the first of these works [51], we study experimental measures of neuroprotective capacity of new 1,3-rasagiline derivatives. All the compounds, except one of them, had a high protective activity against damage mediated by H_2_O_2_. The best one of all, a monopropargyl *trans* derivative, showed also a high neuroprotective action in all three type of assays. Our first model predicted for this compound high probability of activity in relationship with acetylcholine and GABA, in addition to GluRs. In coincidence, acetylcholine receptors (AChRs) have been associated with neuroprotective proprieties in several recent experimental works, and there are also reports of association of GABA and GluRs with neuroprotective ability [70,71]. Nuritova *et al*. [72], discussed a neuroprotective strategy involving retrograde release of glutamate.

In our second work [52], we studied two types of substituent groups (propargyl groups attached to the nitrogen and a carbamate or esther group instead of hydroxyl). The compounds also presented two different chirality patterns but with 1,3 substitutions pattern. The compounds of this second series were active experimentally in the absence and presence of neurotoxic agents. The best compound of this second series, a dipropargyl derivative, was predicted to have brain nitric oxide synthase (bNOS) as the most probable target and certain probability of multi-target ligand. Again, bNOS was associated experimentally with neuroprotective action in several works [73,74].

The compounds studied this third work present similar substituent groups and stereochemistry but one 1,2 substitution pattern. Based on the previous results, we should expect a similar experimental activity and predictions. However, in the previous section we shown experimentally that the present set of compounds seems not to be very active over GluRs and the model predicts the higher scores of activity over 5-HT3Rs instead of the expected receptors. As we stated in the previous paragraph, 5-HT3Rs have been related to neuroprotective properties *in vitro* and *in vivo* [66]. A plausible hypothesis (pendent of further experimental confirmation) is the variation in receptor affinity (from GluRs to 5-HT3Rs pathway) due to the change from 1,3 to 1,2 substitution pattern. From our point of view, these correspondences between targets that our equations predict, and the references cited from the literature could indicate biological plausibility of our models.

## 3. Materials and Methods

### 3.1. Computational Methods

#### 3.1.1. ALMA-Entropy Models

ALMA models may be classified as a general type of model to assessing the links in different systems. They are adaptable to all molecular descriptors and/or graphs invariants or descriptors for complex networks. In general, we refer to a descriptor *D*^i^_k_ of type k^th^ of the i^th^ system (compound or drug *d*_i_ in this case) represented by a matrix **M**. In fact, in this work we are going to compare the model based on entropy values θ^i^_k_ of a Markov matrix ^1^**Π** with other ALMA models based on other invariants of the same matrix ^1^**Π**, or invariants of the bond adjacency matrix ^1^**B**. Consequently, we describe first the general equations of the model using a generic descriptor, or graph theoretical invariant *D*^i^_k_, and later we give the specific equation for the entropy model based on θ^i^_k_ values. The aim of this model is to link the scores *S*_ij_(*c*_q_) with the molecular descriptors *D*^i^_k_ of a given compound *d*_i_ and the Box–Jenkins MA operators written in the form of deviation terms Δ*D*^i^_k_(*c*_q_) = *D*^i^_k_ – <*D*^i^_k_(*c*_q_)>. The model has the following general form:

(5) Sij(cq)=​a0+∑k=0k=5′ak⋅′Skq+∑q=1q=5′′ajk⋅′′Sijk(cq)=​a0+∑k=0k=5′ak⋅p(cl)⋅Dik+∑q=1q=5∑k=0k=5′′ajk⋅ΔDik(cq)​= a0+∑k=0k=5′ak⋅p(cl)⋅Dik+∑q=1q=5∑k=0k=5′′aqk⋅[Dik−p(cq)⋅〈Dik(cq)〉]

The output dependent variable is *S*_ij_(*c*_q_) = *S*_ij_(*c*_l_, *c*_2_, *c*_3_, *c*_4_, *c*_5_) = *S*_ij_(*c*_l_, *a*_q_, *o*_t_, *t*_j_, *s*_x_). The variable *S*_ij_(*c*_q_) is a numerical score of the biological activity of the i^th^ drug (*d*_i_) *vs*. the j^th^ target measured in one assay carried out under the set of q^th^ conditions *c*_q_. Our hypothesis is H_0_: we can calculate the output *S*_i_(*c*_q_) as a linear combination of scores. We have two types of scores. The first type are the scores '*S*_i_*^k^ =* '*a*_k_·*p*(*c*_l_)·^i^*D*_k_ that account for the quality of data *p*(*c*_l_) and for contributions of the k^th^ molecular descriptors to the final activity score *S*_ij_(*c*_q_). In fact, we used the probability *p*(*c*_1_) = 1.0; 0.75; or 0.5 for data curated in CHEMBL database at levels of expert, intermediate, or auto-curation level, respectively. The second type are scores ''*S*_ij_^k^(*c*_q>1_) = ''*a*_k_·Δ*D*^i^_k_(*c*_j_) for the contributions of deviations Δ*D*^i^_k_(*c*_q_) = (*D*^i^_k_ − <*D*^i^_k_(*c*_q_)>) of the descriptors of *d*_i_ from the average of those of active molecules *L*_ij_(*c*_q_) = 1 for different *c*_q_. In general, *c*_j_ refers to different Multi-output assay conditions, e.g., targets, assays, cellular lines, organisms, organs, *etc*. In this sense, *c*_0_ = is the accuracy of the data for this assay, *c*_1_ = *a*_u_ is the assay *per se*, *c*_2_ = *o*_t_ is the organism that express the target, *c*_3_ = *t*_j_ is the j^th^ cellular or molecular target, and *c*_5_ = *s*_x_ is standard experimental measure of activity. Then, the parameter *D*^i^_k_ and Δ*D*^i^_k_(*c*_q_) are the input independent variables and *L*_ij_(*c*_q_) = 1 is the input dependent variable. Here, <*D*^i^_k_(*c*_q_)> is the average of the k^th^ descriptors *D*^i^_k_ of all i^th^ compounds considered as active (*L*_ij_(*c*_q_) = 1) in an assay carry out under the set of conditions *c*_q_. The parameters Δ*D*^i^_k_(*c*_q_) are similar to the MA used in time series analysis for Bob–Jenkins ARIMA models and others [42]. This type of MA model has been used before to solve different problems in Cheminformatics before. It means that, firstly, we sum the values of *D*^i^_k_ for all the *n*_j_ drugs with *L*_ij_(*c*_q_) = 1 in the assay carry out in the conditions *c*_j_. Next, we divide this sum by the number of compounds *n*_j_ with this condition.


(6) 〈Dik(cq)〉=1nq∑i=1i=nqDik(cq)

In this model, we used only one molecular descriptor θ^i^_5_. This is the Shannon entropy of order *k =* 5 calculated with MI. We do not use low-order entropies *k =* 0, 1, 2, 3, and 4. Accordingly, the general equation is:

(7) Sij(cq)=​a0+′a5⋅′Si5(cl)+∑q=1q=5′′ajk⋅′′Sij5(cq)=​a0+′a5⋅p(cl)⋅θi5+∑q=1q=5′′ajk⋅Δθi5(cq)​= a0+′ak⋅p(cl)⋅θi5+∑q=1q=5′′aqk⋅[θi5−p(cq)⋅〈θi5(cq)〉]

This type of moving average or deviation-like models was coined by us as the ALMA models, and has been used before to solve different problems [54,75,76,77]. In order to seek the model we used the technique Linear Discriminant Analysis (LDA) implemented in the software package STASTICA 6.0 [78]. The statistical parameters used to corroborate the model were: Number of cases in training (*N*), and overall values of Sp, Sn, and Ac [54].

#### 3.1.2. CHEMBL Dataset

We downloaded from the public database CHEMBL a general data set composed of >8000 Multi-output assay endpoints (results of multiple assays) [33,34]. We assigned a value of the observed (obs) class variable *L*_ij_(*c*_q_)_obs_ = 1 (active compound) or *L*_ij_(*c*_q_)_obs_ = 0 (non-active compounds) to every i^th^ drug biologically assayed in different conditions *c*_j_. The dataset used to train and validate the model includes *N* = 3548 statistical cases, formed by *N*_d_ = 3091 unique drugs which have been assayed each one in at least one out of 37 possible standard type measures determined in, at least, one out of 493 assays. Each assay involves, in turn, at least one out of 169 molecular or cellular targets expressed in the tissues of at least one out of 11 different organisms (including human).

### 3.2. Experimental Methods: Chemistry

#### 3.2.1. Synthesis of 1,2-Rasagiline Derivatives

Melting points are uncorrected and were determined in Reichert Kofler Thermopan (Reichert, Vienna, Austria) or in capillary tubes on a Büchi 510 apparatus (BÜCHI Labortechnik AG, Flawil, Switzerland). Infrared spectra were recorded on a JASCO FT/IR-4100 spectrophotometer (JASCO Analytical Instruments, Easton, PA, USA). The ^1^H-NMR spectra (300 MHz) and ^13^C-NMR spectra (75 MHz) were recorded in a Bruker AMX spectrometer (Bruker BioSpin Corporation, Fremont, CA, USA), using TMS as internal reference (chemical shifts in δ values, *J*. in Hz). EI Mass spectra were recorded on a HEWLETT-PACKARD 5988A spectrometer (Hewlett-Packard Company, Palo Alto, CA, USA). FABMS were obtained using MICROMASS AUTOSPEC mass spectrometer (WATERS, Milford, MA, USA) and ESIMS were determined on a BRUKER AMAZON ETD spectrometer (Bruker BioSpin Corporation). We performed microanalyses in a Perkin-Elmer 240B elemental analyzer (PerkinElmer, Waltham, MA, USA) by the Microanalysis Service of the University of Santiago de Compostela. The specific rotation was measured with a PERKIN-ELMER 241 polarimeter (PerkinElmer), and it is expressed in (°) (dm^−1^) (g^−1^) (mL). Most of the reactions were monitored by TLC on pre-coated silica gel plates (Merck 60 F254, 0.25 mm, Merck KGaA, Darmstadt, Germany). Synthesized products were purified by flash column chromatography on silica gel (Merck 60, 230–240 mesh, Merck KGaA) and crystallized if necessary. Solvents were dried by distillation prior use.

Compound (**3**): (1*S*,2*R*)-(+)-*cis*-1-(*N*-Propargylamino)-2-indanol (2) and (*1S*,2*R*)-(+)-*cis*-1-(*N*,*N*-dipropargylamino)-2-indanol. A mixture of **1** (0.20 g, 1.34 mmol), K_2_CO_3_ (0.18 g, 1.34 mmol) and MeCN (7 mL) was stirred at room temperature under argon for 5 min. A solution of propargyl bromide (0.3 mL, 2.7 mmol) dissolved in MeCN (2 mL) was added dropwise with stirring. After being stirred for 24 h, the solvent was evaporated and the residue was dissolved in EtOAc (10 mL). The organic layer was washed with NaOH 2N (3 × 10 mL) and dried (Na_2_SO_4_). The removal of excess of solvent to give a white solid, that was purified by flash column chromatography using hexane/EtOAc (3:1) as eluent to give, in first place **3** (170 mg, yield 56%) as a white solid and in second place **2** (90 mg, yield 36%) as a white solid.

(+)-*cis*-**2**. M.p. 106–108 °C. ${\left[\propto \right]}_{D}^{25ºC}$ = +38° (25 °C, 0.25, CHCl_3_). IR ν = 3277, 2906, 1421, 1339, 1140, 1051, 731 cm^−1^. ^1^H NMR (300 MHz, CDCl_3_) δ = 7.32–7.22 (m, 4H, H_arom_), 4.51–4.47 (m, 1H, 2-H), 4.31–4.29 (m, 1H, 1-H), 3.69–3.52 (AB system , 1H, *J* = 17.2 Hz, CH_2_), 3.68–3.51 (AB system, 1H, *J*. *=* 17.2 Hz, CH_2_), 3.11–2.96 (m, 2H, 3α-H, 3β-H), 2.67 (br. s., 1H, D_2_O exch., OH), 2.31 (t, 1H, *J*. = 2.2 Hz, CH). ^13^C RMN (75 MHz, CDCl_3_) δ = 141.85 (C-3a), 141.05 (C-7a), 128.17, 126.79, 125.58 and 123.94 (CH_arom_), 82.27 (C≡CH), 71.90 (C-2), 70.87 (C≡CH), 64.78 (C-1), 39.59 (CH_2_), 37.16 (C-3). MS (EI): *m*/*z* (%): 186 (2) [M−1]^+^, 168 (5) [M^+^–H_2_O], 148 (100) [M^+^–propargyl], 130 (21), 115 (10), 103 (31), 77 (11). Anal. calcd. for C_12_H_13_NO (187.24): C 76.98, H 7.00, N 7.48; found C 76.63, H 7.12, N 7.36.

(+)-*cis*-**3**. M.p. 106–109 °C. ${\left[\propto \right]}_{D}^{25ºC}$ = +72° (25 °C, 0.25, CHCl_3_). IR ν = 3279, 2894, 1339, 1244, 1137 cm^−1^. ^1^H NMR (300 MHz, CDCl_3_) δ = 7.52–7.50 (m, 1H, 7-H), 7.29–7.18 (m, 3H, 4-H, 5-H, 6-H), 4.52 (dd, 1H, *J*. = 13.2, 6.9 Hz, 2-H), 4.42–4.40 (m, 1H, 1-H), 3.74 (br. s., 1H, D_2_O exch., OH), 3.65–3.39 (AB system, 2H, *J*. = 17.1 Hz, CH_2_), 3.64–3.38 (AB system, 2H, *J*. = 17.1 Hz, CH_2_), 3.24–2.79 (part AB of an ABM system, 2H, *J*_AB_ = 16.4 Hz, *J*_AM_ =7.2 Hz, *J*_BM_ = 6.1 Hz, 3α-H, 3β-H), 2.29 (t, 2H, *J*. = 2.3 Hz, 2 × CH). ^13^C RMN (75 MHz, CDCl_3_) δ = 141.47 (C-3a), 138.15 (C-7a), 128.68, 127.04, 126.65 and 125.46 (CH_arom_), 80.37 (2 × C≡CH), 72.87 (C-2), 71.39 (2 × C≡CH), 68.37 (C-1), 41.04 (2 × CH_2_), 40.31 (C-3). MS (EI): *m*/*z* (%): 226 (2) [M+1]^+^, 225 (5) [M^+^], 224 (4) [M−1]^+^, 208 (2) [M^+^–H_2_O], 186 (100) [(M−1)^+^–propargyl], 133 (32), 116 (35), 77 (29). Anal. calcd. for C_15_H_15_NO (225.29): C 79.97, H 6.71, N 6.22; found C 79.81, H 6.92, N 6.29.

Compound (**4**): (1*S*,2*R*)-(−)-*cis*-1-(*N*,*N*-Dipropargylamino)-2-indanyl acetate. A mixture of **3** (0.08 g, 0.36 mmol), acetic anhydride (66 μL, 0.72 mmol), Et3N (100 μL, 0.72 mmol), DMAP (a catalytic amount) in MeCN (5 mL), under argon, was stirred at room temperature for 3 h. The solvent was removed and the residue was partitioned between EtOAc (10 mL) and H2O (10 mL), and the organic layer was washed with a saturated solution of NaCl (3 × 10 mL), dried (Na2SO4) and evaporated, to give **4** (as a white solid (76 mg, yield 80%). M.p. 52–53 °C. ${\left[\propto \right]}_{D}^{25ºC}$ = −70.6° (25 °C, 0.25, CHCl_3_). IR ν = 3239, 2890, 1729, 1210, 1035 cm^−1^. ^1^H NMR (300 MHz, CDCl_3_) δ = 7.49–7.46 (m, 1H, 7-H), 7.31–7.21 (m, 3H, 4-H, 5-H, 6-H), 5.68 (dt, 1H, *J*. = 5.4, 2.4 Hz, 2-H), 4.61 (d, 1H, *J*. = 5.4 Hz, 1-H), 3.77–3.63 (AB system, 2H, *J*. = 17.5 Hz, CH_2_), 3.76–3.62 (AB system, 2H, *J*. = 17.3 Hz, CH_2_), 3.17–2.93 (part AB of an ABM system, 2H, *J*_AB_ = 17.2 Hz, *J*_AM_ = 5.7 Hz, *J*_BM_ = 2.5 Hz, 3α-H, 3β-H), 2.21 (t, 2H, *J*. = 2.4 Hz, 2 × CH), 2.02 (s, 3H, CH_3_). ^13^C NMR (75 MHz, CDCl_3_) δ = 170.40 (COCH_3_), 140.01 (C-3a), 139.48 (C-7a), 128.07, 126.97, 125.24 and 125.13 (CH_arom_), 81.21 (2 × C≡CH), 77.04 (C-2), 71.74 (2 × C≡CH), 68.94 (C-1), 39.99 (2 × CH_2_), 37.68 (C-3), 21.70 (CH_3_). MS (FAB): *m*/*z* (%): 269 (6) [M+2]^+^, 268 (26) [M+1]^+^, 225 (2) [M^+^–acetyl], 197 (18), 169 (12), 154 (88), 137 (100). Anal. calcd. for C_17_H_17_NO_2_ (267.32): C 76.38, H 6.41, N 5.24; found C 76.12, H 6.68, N 5.36.

Compound (**5**): (1*S*,2*R*)-(−)-*cis*-1-(*N*,*N*-Dipropargylamino)-2-indanyl benzoate. To a solution of of **3** (0.08 g, 0.36 mmol), DMAP (a catalytic amount) in MeCN (5 mL), at 0 °C and under argon, was added dropwise a solution of benzoyl chloride (82 μL, 0.72 mmol) and Et3N (100 μL, 0.72 mmol). The mixture was stirred at room temperature for 2 h. the solvent was evaporated and the residue was dissolved in CH2Cl2 (10 mL). The layer organic was washed with a saturated solution of NaCl (3 × 10 mL), dried (Na2SO4) and evaporated, to give a yellow oil that was purified by flash column chromatography using hexane–EtOAc (6:1) as eluent to give **5** (73 mg, yield 73%) as a yellow oil. ${\left[\propto \right]}_{D}^{25ºC}$ = −85.6° (25 °C, 0.25, CHCl_3_). IR ν = 3289, 2842, 1714, 1267, 1108, 1069 cm^−1^. ^1^H NMR (300 MHz, CDCl_3_) δ = 7.95–7.92 (m, 2H, 2'-H, 6'-H), 7.56–7.50 (m, 7H, 3'-H, 4'-H, 5'-H, 4 × H_arom_), 6.00 (dt, 1H, *J*. = 5.6, 2.6 Hz, 2-H), 4.74 (d, 1H, *J*. = 5.3 Hz, 1-H), 3.75 (d, 4H, *J*. = 2.3 Hz, 2 × CH_2_), 3.29–3.06 (part AB of an ABM system, 2H, *J*_AB_ = 17.0 Hz, *J*_AM_ = 5.7 Hz, *J*_BM_ = 2.7 Hz, 3α-H, 3β-H), 2.15 (t, 2H, *J*. = 2.1 Hz, 2 × CH). ^13^C NMR (75 MHz, CDCl_3_) δ = 166.36 (CO), 140.30 (C-3a), 139.81 (C-7a), 133.16 (C'-4), 130.72 (C'-1), 129.83, 128.61, 128.39, 127.28, 125.49 and 125.43 (4 × CH_arom_, 4 × C'-H), 81.30 (2 × C≡CH), 77.75 (C-2), 72.20 (2 × C≡CH), 69.17 (C-1), 40.40 (2 × CH_2_), 38.13 (C-3). MS (FAB): *m*/*z* (%): 331 (11) [M+2]^+^, 330 (40) [M+1]^+^, 231 (68), 186 (3), 154 (95), 137 (100), 105 (25). Anal. calcd. for C_22_H_19_NO_2_ (329.39): C 80.22, H 5.81, N 4.25; found C 80.05, H 6.01, N 4.34.

#### 3.2.2. Reaction of Carbamylation

To a stirred and ice-cooled solution of **2** or **3** (0.43 mmol) in acetonitrile (5 mL) was added the *N*,*N*-dialkylcarbamyl chloride (0.73 mmol), followed by a dropwise addition of NaH (60% in oil, 0.56 mmol). The reaction mixture was stirred for 24 h at room temperature under argon. After evaporation of the solvent *in vacuo*, water (10 mL) was added and extracted with ether (3 × 10 mL). The organic phase was washed with dilute KOH (pH 10–11), dried and evaporated to dryness *in vacuo*. Purification by column chromatography (Hexane:EtOAc 4:1) afforded:

Compound (**6**): (1*S*,2*R*)-(−)-*cis*-1-(*N*-Propargylamino)-2-indanyl dimethylcarbamate. This compound was obtained as a yellow solid (100 mg, yield 73%). M.p. 119–122 °C. ${\left[\propto \right]}_{\text{D}}^{23\text{ºC}}$ = −50.4° (23 °C, 0.25, CHCl_3_). IR ν = 3264, 2923, 1693, 1388, 1184, 1047 cm^−1^. ^1^H NMR (300 MHz, CDCl_3_) δ = 7.40–7.39 (m, 1H, 7-H), 7.28–7.13 (m, 3H, 4-H, 5-H, 6-H), 5.52–5.48 (m, 1H, 2-H), 4.38 (d, 1H, *J* = 5.0 Hz, 1-H), 3.61–3.46 (AB system, 1H, *J*. = 16.8 Hz, CH_2_), 3.60–3.45 (AB system, 1H, *J*. = 16.8 Hz, CH_2_), 3.19–3.04 (AB system, 1H, *J* = 16.5 Hz, 3α-H), 3.17–3.03 (AB system, 1H, *J*. = 16.5 Hz, 3β-H), 2.90–2.80 (m, 6H, 2 × CH_3_), 2.62 (t, 1H, *J*. = 2.5 Hz, CH), 2.25 (br. s., 1H, D_2_O exch., NH). ^13^C NMR (75 MHz, CDCl_3_) δ = 155.97 (CO), 142.09 (C-3a), 139.75 (C-7a), 127.97, 126.69, 124.93 and 124.66 (CH_arom_), 82.17 (C≡CH), 76.00 (C-2), 71.64 (C≡CH), 63.28 (C-1), 37.46 (CH_2_), 36.35 (C-3), 29.94 and 29.67 (2 × CH_3_). MS (FAB): *m*/*z* (%): 258 (1) [M]^+^, 257 (6) [M−1]^+^, 168 (100), 116 (80), 72 (80). Anal. calcd. for C_15_H_18_N_2_O_2_ (258.32): C 69.74, H 7.02, N 10.84; found C 69.65, H 7.13, N 10.93.

Compound (**7**): (1*S*,2*R*)-(−)-*cis*-1-(*N*-Propargylamino)-2-indanyl diethylcarbamate. Isa yellow solid (98 mg, yield 66%). M.p. 68–69 °C. ${\left[\propto \right]}_{\text{D}}^{23\text{ºC}}$ = −37.6° (23 °C, 0.25, CHCl_3_). IR ν = 3242, 2972, 1677, 1425, 1270, 1173, 1066 cm^−1^. ^1^H NMR (300 MHz, CDCl_3_) δ = 7.42–7.39 (m, 1H, 7-H), 7.29–7.21 (m, 3H, 4-H, 5-H, 6-H), 5.54 (dt, 1H, *J*. = 5.3, 3.6 Hz, 2-H), 4.41–4.39 (m, 1H, 1-H), 3.63–3.49 (AB system, 1H, *J*. = 16.8 Hz, CH_2_), 3.62–3.48 (AB system, 1H, *J*. = 16.8 Hz, CH_2_), 3.29–3.09 (m, 6H, 3α-H, 3β-H, 2 × CH_2_CH_3_), 2.25 (t, 1H, *J*. = 2.4 Hz, CH), 1.93 (br. s., 1H, D_2_O exch., NH), 1.28–1.01 (m, 6H, 2 × CH_2_CH_3_). ^13^C NMR (75 MHz, CDCl_3_) δ = 155.18 (CO), 142.24 (C-3a), 139.80 (C-7a), 127.91, 126.65, 124.88 and 124.61 (CH_arom_), 82.19 (C≡CH), 75.64 (C-2), 71.54 (C≡CH), 63.52 (C-1), 41.92 and 41.30 (2 × CH_2_CH_3_), 37.45 (CH_2_), 36.50 (C-3), 13.99 and 13.51 (2 × CH_2_CH_3_). MS (FAB): *m*/*z* (%): 288 (18) [M+2]^+^, 287 (100) [M+1]^+^, 286 (8) [M]^+^, 285 (6) [M−1]^+^, 231 (21), 154 (27), 137 (26). Anal. calcd. for C_17_H_22_N_2_O_2_ (286.37): C 71.30, H 7.74, N 9.78; found 71.12, H 7.99, N 9.92.

Compound (**8**): (1*S*,2*R*)-(−)-*cis*-1-(*N*,*N*-Dipropargylamino)-2-indanyl dimethylcarbamate. Was obtained as a white solid (76 mg, yield 58%). M.p. 109–112 °C. ${\left[\propto \right]}_{\text{D}}^{25\text{ºC}}$ = −38° (25 °C, 0.25, CHCl_3_). IR ν = 3292, 2922, 1685, 1397, 1272, 1186, 1050 cm^−1^. ^1^H NMR (300 MHz, CDCl_3_) δ = 7.48 (t, 1H, *J* = 3.9 Hz, 7-H), 7.27–7.22 (m, 3H, 4-H, 5-H, 6-H), 5.61 (dt, 1H, *J*. = 5.6, 3.3 Hz, 2-H), 4.63–4.61 (m, 1H, 1-H), 3.67–3.66 (m, 4H, 2 × CH_2_), 3.16–2.97 (AB system, 1H, *J*. = 16.8 Hz, 3α-H), 3.14–2.96 (AB system, 1H, *J*. = 16.8 Hz, 3β-H), 2.91–2.81 (m. 6H, 2 × CH_3_), 2.21 (t, 1H, *J*. = 2.2 Hz, 2 × CH) ^13^C NMR (75 MHz, CDCl_3_) δ = 155.98 (CO), 140.05 (C-3a), 139.82 (C-7a), 127.96, 126.76, 125.39 and 125.10 (CH_arom_), 81.10 (2 × C≡CH), 77.36 (C-2), 71.91 (2 × C≡CH), 68.31 (C-1), 40.24 (2 × CH_2_), 37.94 (C-3), 36.49 and 36.14 (2 × CH_3_). MS (FAB): *m/z* (%): 298 (19) [M+2]^+^, 297 (100) [M+1]^+^, 296 (4) [M]^+^, 295 (9) [M−1]^+^, 231 (30), 204 (21), 154 (31), 137 (39). Anal. calcd. for C_18_H_20_N_2_O_2_ (296.36): C 72.95, H 6.80, N 9.45; found 72.78, H 7.01, N 9.53.

Compound (**9**): (1*S*,2*R*)-(−)-*cis*-1-(*N*,*N*-Dipropargylamino)-2-indanyl diethylcarbamate. This compound was obtained as an oil (70 mg, yield 49%). ${\left[\propto \right]}_{\text{D}}^{25\text{ºC}}$ = −18.6° (25 °C, 0.25, CHCl_3_). IR ν = 3292, 2928, 1688, 1425, 1270, 1167, 1062 cm^−1^. ^1^H NMR (300 MHz, CDCl_3_) δ = 7.49 (t, 1H, *J* = 4.2 Hz, 7-H), 7.28–7.23 (m, 3H, 4-H, 5-H, 6-H), 5.59 (dt, 1H, *J*. = 5.8, 3.9 Hz, 2-H), 4.62 (d, 1H, *J*. = 5.8 Hz, 1-H), 3.71–3.57 (m, 4H, 2 × CH_2_), 3.35–3.23 (m, 4H, 2 × CH_2_CH_3_), 3.17–2.98 (AB system, 1H, *J*. = 16.8 Hz, 3α-H), 3.15–2.97 (AB system, 1H, *J* = 16.8 Hz, 3β-H), 2.21 (t, 2H, *J*. = 2.2 Hz, 2 × CH), 1.12–1.01 (m, 6H, 2 × CH_2_CH_3_). ^13^C NMR (75 MHz, CDCl_3_) δ = 155.21 (CO), 139.98 (C-3a), 139.86 (C-7a), 128.00, 126.74, 125.55 and 125.01 (CH_arom_), 81.06 (2 × C≡CH), 76.58 (C-2), 71.97 (2 × C≡CH), 67.99 (C-1), 41.65 and 41.12 (2 × CH_2_CH_3_), 40.14 (2 × CH_2_), 37.81 (C-3), 13.97 and 13.47 (2 × CH_2_CH_3_). MS (FAB): *m*/*z* (%): 326 (20) [M+2]^+^, 325 (92) [M+1]^+^, 324 (2) [M]^+^, 323 (8) [M−1]^+^, 288 (89), 230 (51), 154 (71), 137 (100). Anal. calcd. for C_20_H_24_N_2_O_2_ (324.42): C 74.04, H 7.46, N 8.64; found 73.89, H 7.61, N 8.75.

### 3.3. Experimental Methods: Biology

#### 3.3.1. Culture of Rat Cortical Neurons

Embryos were selected from 19 to 20 days pregnant rats by caesarean section. Meninges were removed and cortex was isolated after the dissection of the brain. The fragments obtained from several embryos were subjected to mechanic digestion. We re-suspended the cells in a Neurobasal medium with 2% B-27. We seeded in 48-well plates at a density of 100,000 cells/mL. Neuronal cultures were allowed to grow for 8–10 days. Incubations with different CSF were done when the microscope showed the existence of a dense neuronal network. Embryos were selected from 19 to 20 days pregnant rats, which were decapitated and embryos were extracted from the womb by caesarean section. Meninges were removed and a portion of motor cortex was isolated after the dissection of the brain. Fragments obtained from several embryos were subjected to mechanic digestion and cells were re-suspended in Neurobasal medium with 2% B-27 and seeded in 48-well plates at a density of 100,000 cells/mL. Neuronal cultures were allowed to grow for 8–10 days and when the microscope showed the existence of a dense neuronal network, incubations with different CSF were done [79].

#### 3.3.2. Measurement of Neuronal Viability

We used the MTT reduction assay following the procedure previously described [65]. After the appropriate incubations with the compounds alone, or co-incubated with 100 µM H_2_O_2_ or glutamate, 0.5 mg/mL MTT were added to each well and incubation was performed at 37 °C for 2 h. Formazan salt formed was dissolved in DMSO, and colorimetric determination were performed at 540 nm. Control cells without compounds or toxic stimulus were considered 100% viability. Neuronal viability after exposure to compounds or different treatments was expressed as% of control within each individual experiment. Graph Pad Prism Software (GraphPad Software, San Diego, CA, USA) was used to perform statistical analyses and graphical presentation. Experiments were reproduced at least three times. Data were expressed as mean ± S.E.M. values. Groups were compared by ANOVA/Dunnett’s test. A *p-*value ≤0.05 was accepted as the limit of statistical significance.

## 4. Conclusions

We can use Shannon entropy measures to developing predictive models for multi-target networks of neuroprotective/neurotoxic compounds. In doing so, we can use Box–Jenkins operators of molecular descriptors to obtain multi-target, multi-scale, and multi-output models able to predict different outcomes for multiple combinations of output experimental measures, experimental protocols, organisms, and molecular and cellular targets. One of these models has been demonstrated here to be useful as a complementary tool in the organic synthesis and evaluation of the multi-target biological activity of new compounds with potential neuroprotective activity. The model is also a very useful tool to predict complex networks of drug-target interactions with possible applications to the study of non-linear effects in the biological activity of neuroprotective drugs.

## Supplementary Information

Supplementary material files which contain detailed lists of the values of parameters are available upon requests to the corresponding author.
